# Analysis of Volatile Compounds in Soju, a Korean Distilled Spirit, by SPME-Arrow-GC/MS

**DOI:** 10.3390/foods9101422

**Published:** 2020-10-08

**Authors:** Jiyoon Cha, Young-Wook Chin, Jun-Young Lee, Tae-Wan Kim, Hae Won Jang

**Affiliations:** Korea Food Research Institute, 245 Nongsaengmyeong-ro, Iseo-myeon, Wanju-Gun, Jeollabuk-do 55365, Korea; Chajiyoon@kfri.re.kr (J.C.); ywchin@kfri.re.kr (Y.-W.C.); j.y.lee@kfri.re.kr (J.-Y.L.)

**Keywords:** volatile compounds, volatile extraction, solid-phase microextraction arrow, gas chromatography–mass spectrometry, Korean distilled spirits (Soju)

## Abstract

The SPME Arrow technology—a novel solid phase micro-extraction technique—was used to analyze Soju, a traditional Korean distilled liquor, in barrels made of *Quercus* spp. The volatile compounds detected when the barrels were toasted were analyzed. Five types of sorbents—carbon wide range/polydimethylsiloxane, divinylbenzene/carbon wide range/polydimethylsiloxane, divinylbenzene/polydimethylsiloxane, polydimethylsiloxane, and polyacrylate—were used for this investigation. Fifty-four volatile compounds were detected in Soju using gas chromatography/mass spectrometry. A high extraction efficiency was obtained using carbon wide range/polydimethylsiloxane. Nineteen samples were analyzed using barrels made of six species of carbonated oak (*Q. aliena*, *Q. variabilis*, *Q. dentate*, *Q. acutissima*, *Q. mongolica*, and *Q. serrata*) and control groups in three ways: noncharring, medium charring, and heavy charring. Ethanol, 1-propanol, isoamyl acetate, and isoamyl alcohol can be used as indicator volatile components for Soju and other such traditional Korean distilled liquors. We believe our study results can be used to design better analysis methods for Soju and other distilled liquors.

## 1. Introduction

Profiling of volatile compounds is an important step in determining the flavor of distilled spirits. Volatile compounds form during fermentation, distillation, and storage of distilled spirits, and are affected by various factors such as the type of raw material and the manufacturing and aging processes [1,2]. Soju, a traditional liquor of Korea, is aged in oak barrels after distillation from steamed rice or mixed grains and Nuruk [3]. The quality of Soju is greatly influenced by its volatile component [4].

Wines are typically stored in oak barrels. However, it causes their oxidation owing to the continuous flow of a small amount of air through a bunghole in the barrels; as a result, new volatile compounds are produced in the wine during aging [5,6]. It has been reported that there are significant differences in the scents of different oak species [7,8,9]. In the past, red pine used to be widely distributed in Korea; however, the area under red pine has decreased in recent times owing to climate change. On the other hand, *Quercus* spp., a dominant broad-leafed species, is gradually spreading in the country [10]. To produce high-quality Korean distilled spirits, the following six species of Korean oak should be used: Galchamnamu (*Q. aliena*, GAL), gulchamnamu *(Q. variabilis*, GUL), tteoggalnamu (*Q. dentate*, DUK), sangsurinamu (*Q. acutissima*, SAN), singalnamu (*Q. mongolica*, SIN), and jolchamnamu (*Q. serrata*, JOL). These oaks also are part of broad-leafed Korean forests and were selected as representative oak species of Korea.

The conventional extraction methods for analyzing volatile food substances are vacuum simultaneous steam distillation solvent extraction (V-SED) [11,12], direct solvent extraction (DSE) [13,14], solvent-assisted flavor evaporation (SAFE) [15], and steam distillation under reduced pressure (DRP-LLE) [16,17]. However, these methods need large amounts of extraction solvents, have low recovery, and can be used only with low-fat samples. To offset these disadvantages, headspace extraction methods such as dynamic headspace (DHS) [18], headspace solid-phase microextraction (HS-SPME) [19], and headspace stir-bar sorptive extraction (HS-SBSE) [20,21] are widely used. Headspace extraction methods do not need organic solvents and they can be applied with even small amounts of samples. Moreover, their selectivity and extraction efficiency can be improved by using a quick sorbent that does not take long to extract [22,23,24,25].

Recently, a new technology—SPME Arrow—has been developed that combines the benefits of both SPME and HS-SBSE. SPME Arrow protects the fiber with an outer metal tube, making the fiber more stable and extending its lifetime. As a result, the limitations of mechanical failure associated with SPME can be overcome [26]. In addition, SPME Arrow comprises fibers of greater diameter and length than the SPME fibers [27,28,29], providing a larger amount of the adsorbent phase, affording up to 10 times higher sensitivity [30,31]. In addition, similar to SPME, SPME Arrow can be fully automated. Therefore, SPME Arrow is more advantageous for analyzing volatile substances in various types of foods [32,33].

The present study aimed to find a suitable sorbent for Soju by analyzing the volatile substances present in different Korean distilled spirits using SPME Arrow. Five SPME Arrow fibers were analyzed for optimization. Finding a suitable adsorbent and using the results obtained, the study aims to analyze changes in the volatile profiles of Soju when aged in oak barrels made of six species of carbonated oak. Traditional Korean liquors such as Soju have not been sufficiently studied, in contrast to the distilled liquors of other countries that have been investigated extensively. This study will help improve the analysis of such Korean liquors.

## 2. Materials and Methods

### 2.1. Materials and Reagents

Linalool, used as an internal standard (ISTD), and C7–C40 saturated alkane standard (49452-U, Supelco) were purchased from Sigma-Aldrich (St. Louis, MO, USA). The SPME Arrow fibers were coated with carbon wide range/polydimethylsiloxane (CAR/PDMS, 120 μm × 20 mm), divinylbenzene/carbon wide range/polydimethylsiloxane (DVB/CAR/PDMS, 120 μm × 20 mm), polydimethylsiloxane (PDMS, 120 μm × 20 mm), divinylbenzene/polydimethylsiloxane (DVB/PDMS, 120 μm × 20 mm), and polyacrylate (PA, 100 μm × 20 mm), which were supplied by CTC Analytics AG (Zwingen, Switzerland). The SPME fiber was coated with the carboxen^®^/polydimethylsiloxane (CAR/PDMS, 85 μm) adsorbent (Supelco, Bellefonte, PA, USA). In addition, 20-mL headspace vials and caps with PTFE/silicone septa were purchased from Thermo Fisher Scientific (West Palm Beach, FL, USA).

### 2.2. Manufacture of Distilled Spirits

Here, 1800 kg of white rice (Yeoju rice) was used as a raw material. In addition, 600 kg of rice ipguk was prepared using Baekguk (*Aspergillus luchuensis*, Chungmujongguk). *Saccharomyces cerevisiae* 88-4 strain of Korea Food Research Institute was used as a fermentation starter. The fermentation was carried out in two stages: the first stage was performed for 5 days and the second stage was carried out for 15 days at 25–30 °C. The fermentation yielded 4180 L of liquor with 19.2% (*v*/*v*) alcohol content. The fermented liquor was then distilled under reduced pressure (100 mmHg) and 1548 L of distilled spirit with 49.3% (*v*/*v*) alcohol was obtained.

### 2.3. Manufacture of Korean Oak Casks and Maturation of Distilled Spirits

To investigate the effects of the oak species on flavor compounds of the distilled spirits, five L-scale of casks were made using six species of Korean oaks (see Section 1). Raw woods were provided by the National Institute of Forest Science. Oak casks were manufactured by Chungbuk Youngdong Oak Cask Production Center. Eighteen experimental groups were set up by performing three types of toasting (non-, medium-, and heavy charring) using a gas torch burner to carbonize the inside of the casks. All experiments were performed in duplicate. The “Noncharring” cask had no internal carbonization. In the “Medium-Charring” cask, the inner surface was toasted for approximately 1 min. The “Heavy-Charring” cask was toasted with a fire for approximately 2 min and carbonized until its entire surface became evenly shaped like an alligator skin. Next, 5 L of the distilled spirit was injected into each prepared cask, and maturation was performed at room temperature (20 °C) for 3 months [34,35]. After the maturation, each duplicate sample was homogenized by vatting.

### 2.4. Headspace Solid-Phase Microextraction Arrow Procedure

The HS-SPME Arrow was performed using a Triplus RSH Autosampler (Thermo Fisher Scientific Inc., West Palm Beach, FL, USA) in triplicate. To extract the volatile compounds, 3 mL of the distilled liquor and 75 μg/mL of linalool (200 μg/mL) as ISTD were added into the headspace screw-top vial (20 mL) to achieve the final ISTD concentration of 4.878 μg/mL. The samples were heated and stirred at 40 °C for 10 min at 500 rpm to reach equilibrium. The SPME Arrow fiber was exposed to the headspace for 30 min at 1000 rpm. Then, it was inserted into the GC injector port operating in split mode (5:1) and desorbed at 220 °C for 5 min. After desorption, the Arrow fiber was subjected to a postconditioning treatment for 5 min to eliminate any possible contamination before performing the next analytical run.

### 2.5. Gas Chromatography-Mass Spectrometry Analysis

The analysis was performed using a TRACE 1310 gas chromatograph equipped with a TSQ 9000 triplus quadrupole mass spectrometer (Thermo Fisher Scientific Inc, West Palm Beach, FL, USA). All samples were analyzed using the DB-WAX capillary column (60 m × 0.25 mm i.d. × 0.25 μm film thickness, J&W Scientific Inc., Folsom, CA, USA). Helium gas was used as the carrier gas at a constant flow of 1.0 mL/min. The GC oven programs were held at 45 °C for 10 min, and then first increased to 105 °C at 2 °C/min and then to 220 °C at 3 °C/min and held for 5 min. The ion source and transfer line temperatures were maintained at 230 °C and 280 °C, respectively. The mass range was 35–550 *m*/*z* and the scan rate was 0.4 scan per second in full scan mode. Electron ionization was carried out using ionization energy of 70 eV. The volatile compounds were identified by comparing the mass spectrum ratio of the sample with the data available in Wiley Registry 11th edition/NIST 2017 Mass Spectral Library. The experimental retention index was calculated using alkane standards (C7–C40) and compared with the literature retention index on DB-Wax. KI for the unknown compounds was calculated using the following equation [26]:

$${\mathrm{KI}}_{A}=\;100\;\times \;[n+\;(N\;–\;n)\;\frac{T_{r\left(A\right)}\;-T_{r\left(n\right)}}{T_{r\left(N\right)}\;-T_{r\left(n\right)}}]$$

where:

*KI* = Kováts index of compound A

*n* = number of carbon atoms in the smaller n-alkane eluting before compound A

*N* = number of carbon atoms in the larger n-alkane eluting after compound A

*Tr*(*A*) = retention time of compound A

*Tr*(*n*) = retention time of the smaller n-alkane eluting before compound A

*Tr*(*N*) = retention time of the larger n-alkane eluting after compound A.

The volatile compounds were quantified as relative concentrations calculated by comparing the peak area of each compound with that of the internal standard (linalool, 4.878 μg/L). The results were expressed as the average of triplicate runs for each of the 18 samples.

### 2.6. Statistical Analysis

The mean±standard deviation from triplicate measurements was presented. We assessed the results by analyzing the variance and Duncan’s multiple range test to identify significant differences (*p* < 0.01) using SPSS Statistics ver. 23 (IBM, Armonk, NY, USA). Heatmaps obtained using hierarchical clustering techniques were analyzed and visualized using the MetaboAnalyst version 4.0 software (available at www.metaboanalyst.ca).

## 3. Results

### 3.1. Optimization of SPME Arrow Fibers

**Figure 1:**
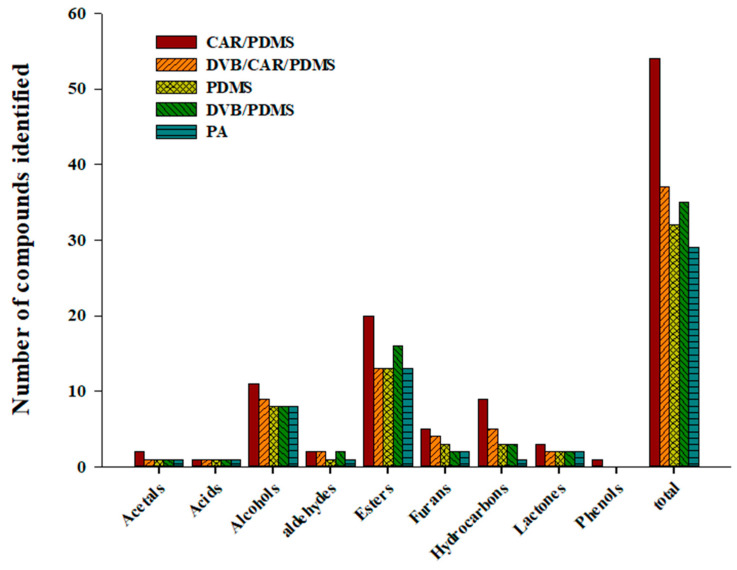
Total number of peaks detected for each class of volatile compounds in Soju (GUL-5) using five types of HS-SPME Arrow fibers (CAR/PDMS, DVB/CAR/PDMS, PDMS, DVB/PDMS, and PA).

The polarity and volatility of the material to be analyzed determined the extraction efficiency of different SPME Arrow fibers [36]. Therefore, an appropriate SPME Arrow fiber was selected for analyzing the volatile compounds of distilled spirits using five commercial sorbents (120 μm CAR/PDMS, 120 μm DVB/CAR/PDMS, 120 μm PDMS, 120 μm DVB/PDMS, and 100 μm PA). Figure 1 shows the total number of peaks detected for each class of volatile compounds in distilled spirits. The Arrow fibers are classified according to their relative polarity. PDMS is relatively nonpolar; CAR/PDMS and DVB/PDMS are semi-polar; and PA and DVB/CAR/PDMS are polar [37]. Fifty-four volatile compounds, the highest number, were identified in the Arrow fiber with the CAR/PDMS sorbent and can be divided in the following nine classes: acetals (2), acids (1), alcohols (11), aldehydes (2), esters (20), furans (5), hydrocarbons (9), lactones (3), and phenols (1). Thirty-seven volatile compounds, the second highest number, were identified in the fiber with the DVB/CAR/PDMS sorbent: acetals (1), acids (1), alcohols (9), aldehydes (2), esters (13), furans (4), hydrocarbons (5), lactones (2), and phenols (0). SPME Arrow fibers with PDMS, DVB/PDMS, and PA could detect 32, 35, and 29 volatile compounds, respectively.

**Figure 2:**
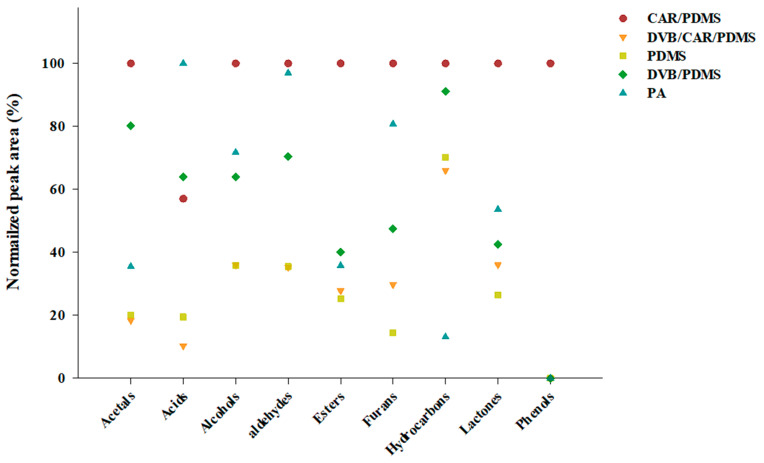
Comparison of the sum of normalized peak areas for each class of volatile compounds in Soju (GUL-5) using five types of SPME Arrow fibers.

The normalized peak areas for each class of the volatile compounds obtained using SPME Arrow fibers with different sorbents were compared (Figure 2). The CAR/PDMS, with a total peak area of 77,745 × 10^6^, showed a high normalized peak area for all the classes of volatile compounds, except for acids. The Arrow fiber with PA showed the highest normalized peak area for the acid class, with a total peak area of 51,206 × 10^6^. The total peak areas observed with DVB/CAR/PDMS, PDMS, and DVB/PDMS were 57,485 × 10^6^, 25,751 × 10^6^, and 46,151 × 10^6^, respectively. The results obtained with the CAR/PDMS fiber showed completely different trends from the other four fibers.

The reproducibility of SPME Arrow and SPME fibers was evaluated to select a suitable fiber type for analyzing the volatile compounds of Soju. SPME Arrow and SPME extraction methods were tested under the same conditions: type of fiber, CAR/PDMS; extraction temperature, 40 °C; extraction time, 30 min; number of runs, triplicate. Supplementary Figure S1 shows the average of relative standard deviation (RSD) and standard deviation of RSD. The SPME Arrow fiber showed the following data: peak area, 7.7414 × 10^10^; the average of RSD, 3.61%; and standard deviation of RSD, 4.71%. The SPME fiber had the following features: peak area, 1.0687 × 10^10^, which is approximately seven times less than that of the SPME Arrow fiber; the average of RSD, 4.41%; and standard deviation of RSD, 9.92%. These results showed that the SPME Arrow fiber can be used for the analysis of volatile compounds in distilled spirits as it had better reproducibility than the SPME fiber.

### 3.2. Volatile Compounds of Soju

**Figure 3:**
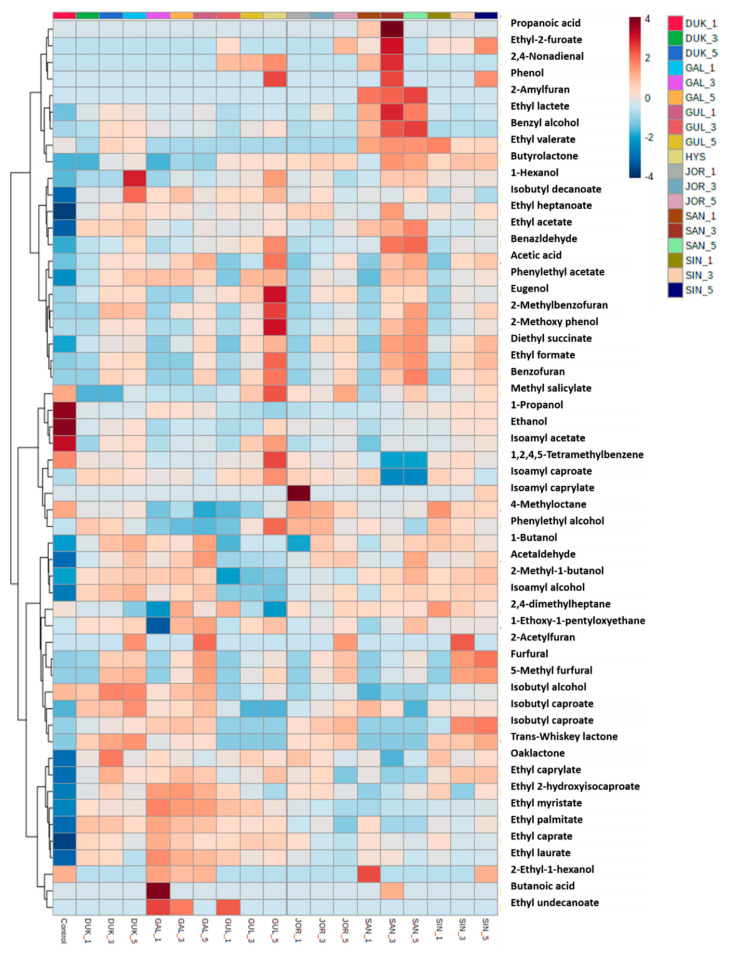
Heatmaps of volatile compounds of Soju analyzed using different types of carbonized Korean oak species.

Based on the above experiment results, CAR/PDMS was used as the adsorbent to perform the experiment. Nineteen samples aged in barrels made of six Korean oak species (GAL, GUL, DUK, SAN, SIN, and JOL) were analyzed. Samples that were not aged were used as controls. These samples were carbonized in three ways: noncharring (1), medium charring (3), and heavy charring (5) (Supplementary Table S1). Acids were detected with a significant result, indicating that their concentration tends to increase with aging and carbonization regardless of the oak species. In the control group, the concentration of acetic acid is 0.68 μg/mL, and the GAL-5 sample had the highest concentration of acetic acid (6.99 μg/mL). Acetic acid has a sour taste, which turns bitter with a vinegar-like scent at high concentrations [38]. Among the alcohols, the concentrations of ethanol and 1-propanol tended to become lower than that of their counterparts in the control. In contrast, the concentrations of 2-methyl-1-butanol, described as malty and chocolaty [39], and isoamyl alcohol, described as having the taste of red fruit and framboise [40], were higher than those of their counterparts in the control. In particular, 2-methyl-1-butanol and isoamyl alcohol were detected at higher concentrations in GUL, DUK, SAN, and SIN samples than in GAL and JOR samples. Twenty-one types of esters were detected, and the concentration of only isoamyl acetate, described as having a fruity, sweet, and banana-like flavor [41], was lower (3.84–7.29 μg/mL in all samples) than that in the control (9.57 μg/mL). However, the esters showed a significant increase in the concentration with aging and carbonization regardless of the oak species used. The concentration of all esters (especially ethyl acetate, ethyl caprylate, ethyl caprate, and phenylethyl acetate), except for isoamyl acetate, increased in all aged samples and became higher than that in the control. Furans, which contain seven volatile compounds, were detected in the samples that underwent carbonization irrespective of the oak species used. However, they were not detected in the control and noncharring samples, and their concentration particularly increased in the samples subjected to medium and heavy charring as these samples were toasted for a longer duration. Furfural and 5-methylfurfural, the major furans, are formed when the wood is baked owing to the breakdown of carbohydrates, and their concentration in Soju depends not only on the age of the barrel, but also on the degree of wood toast [42]. Furfural was described as having a caramel, bready, and cooked meat-like flavor, while 5-methylfurfural was described as having an almond and marzipan-like flavor [43]. The concentration of hydrocarbons tended to become lower than that of the control. Trans-Whiskey lactone and oaklactone were not detected in the control, GAL, and SAN samples, while they were detected in GUL (0.29 μg/mL), DUK (0.22 μg/mL), SIN (0.47 μg/mL), and JOL (0.35 μg/mL), which smell like toasted, woody, coconut, and vanilla [44] and were present in oak wood [45]. Among the phenols, the eugenol concentration tended to increase with aging and carbonization. The sample with the highest concentration of eugenol is GAL-5 (0.27 μg/mL). As shown in Figure 3, heatmaps were plotted in order to reveal the difference in volatile compounds between the samples. A color gradient of dark blue (−4) indicates values below the average, while a color gradient of dark red (4) indicates values above the average. The volatile compounds that showed the most significant difference in their concentration based on the oak species and carbonization were ethanol, 1-propanol, isoamyl acetate, isoamyl alcohol, furfural, and 5-methylfurfural. Furfural and 5-methylfurfural served as indicators whose flavor varies depending on the carbonization type. In addition, 2-methyl-1-butanol, isoamyl alcohol, ethyl acetate, ethyl caprylate, ethyl caprate, and phenylethyl acetate can be used as indicators of aged distilled spirit in oak.

## 4. Conclusions

Volatile components of food have been widely analyzed by the headspace extraction method. In this study, volatile compounds of Soju, a traditional distilled liquor of Korea, were extracted with the SPME Arrow fiber, which shows better robustness and sensitivity than the SPME fiber. The extraction efficiencies of volatile compounds were compared using five types of SPME Arrows. Fifty-four volatile compounds were identified: acetals (2), acids (1), alcohols (11), aldehydes (2), esters (20), furans (5), hydrocarbons (9), lactones (3), and phenols (1). The results showed that volatile compounds are extracted differently based on the properties of the sorbent used with the SPME Arrow fiber. The use of CAR/PDMS afforded high extraction efficiency. Using the results obtained, the volatile components of Soju stored in barrels made of different species of carbonized Korean oak were profiled. Ethanol, 1-propanol, isoamyl acetate, and isoamyl alcohol can be used as indicator volatile components. Our findings support the use of SPME Arrow for analyzing volatile compounds present in food. The results also show that future research into the volatiles of traditional Korean distilled liquors that affect their flavor and taste can be furthered by using different processing methods.

## Supplementary Materials

The following are available online at https://www.mdpi.com/2304-8158/9/10/1422/s1, Figure S1: Reproducibility of CAR/PDMS SPME Arrow and CAR/PDMS SPME fibers measured by comparing their average of RSD (%) and standard deviation of RSD (%), Table S1: Volatile compounds of Soju analyzed using different species of carbonized Korean oak.
