# Supplementary material for: Analysis of Volatile Compounds in Soju, a Korean Distilled Spirit, by SPME-Arrow-GC/MS
Source: Foods. 2020 Oct 8;9(10):1422. doi: 10.3390/foods9101422 (PMC7601619; doi:10.3390/foods9101422)
Supplement: Supplementary file 1 [file foods-09-01422-s001.pdf]

foods MDPI

Supplementary Materials

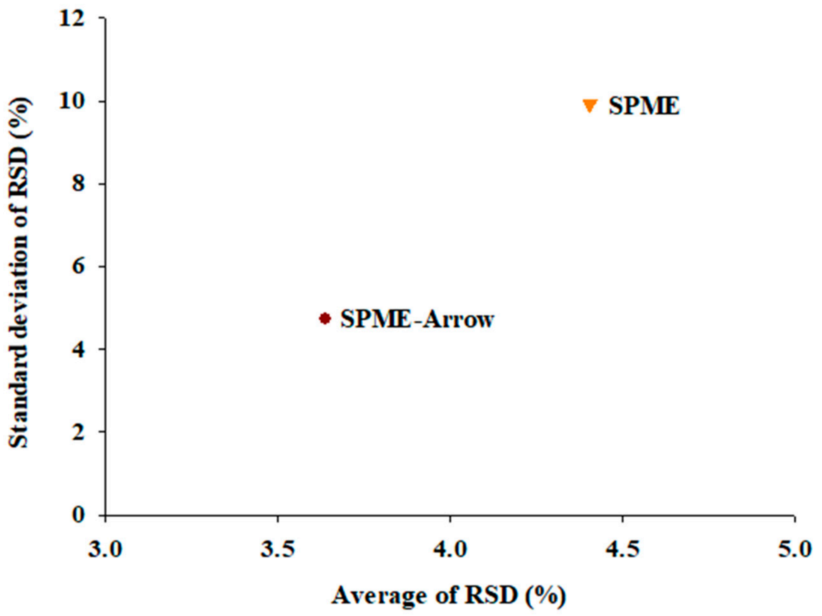

Supplementary Figure 1. Reproducibility of CAR/PDMS SPME Arrow and CAR/PDMS SPME fibers measured by comparing their average of RSD (%) and standard deviation of RSD (%)

Supplementary Table 1. Volatile compounds of Soju analyzed using different species of carbonized Korean oak

Compounds	KI[L]	KI[M]	Relative Concentration (μg/mL) [N]												
			Control	GUL-1	GUL-3	GUL-5	GAL-1	GAL-3	GAL-5	DUK-1	DUK-3	DUK-5	SAN-1	SAN-3	SAN-5
Acetals															
1-Ethoxy-1-pentyloxyethane	1104	1111	0.06±0.01[cd]	ND	0.08±0.01[ab]	0.09±0.01[a]	0.06±0.01[cd]	0.07±0.01[abc]	0.08±0.01[ab]	0.07±0.01[abc]	0.07±0.01[abc]	0.07±0.01[abc]	0.05±0.00[d]	0.06±0.0[bcd]	0.08±0.01[ab]
Acids															
Acetic acid	1461	1461	0.68±0.05[j]	2.94±0.24[efg]	4.44±0.09[cd]	5.50±0.39[bc]	1.04±0.03[hij]	2.30±0.25[gh]	6.99±0.45[a]	2.11±0.36[ghi]	3.72±0.13[def]	3.88±0.55[def]	1.11±0.06[hij]	4.93±0.58[bcd]	5.77±0.45[ab]
Propanoic acid	1556	1559	ND	ND	ND	ND	ND	ND	ND	ND	ND	ND	0.19±0.01[b]	0.64±0.11[a]	ND
Butanoic acid	1663	1658	ND	4.22±0.21[a]	ND	ND	ND	ND	ND	ND	ND	ND	ND	1.66±0.21[b]	ND
Alcohols															
Ethanol	937	941	414.34±34.60[a]	211.98±4.34[e]	228.96±7.09[bcde]	228.83±2.40[bcde]	215.47±9.66[de]	220.66±4.79[cde]	270.50±3.22[b]	235.72±16.83[bcde]	249.15±2.82[bcde]	258.72±3.17[bc]	218.10±11.02[cde]	237.04±3.44[cde]	239.54±14.85[bcde]
1-Propanol	1061	1069	4.10±0.54[a]	1.92±0.14[bc]	1.88±0.02[bc]	1.74±0.08[bcde]	1.34±0.07[efg]	1.23±0.02[fg]	1.09±0.08[g]	1.57±0.06[bcdef]	1.52±0.08[bcdef]	1.53±0.07[bcdef]	1.42±0.14[defg]	1.51±0.04[cdefg]	1.75±0.22[bcde]
Isobutyl alcohol	1108	1119	20.78±0.44[abc]	18.55±1.61[cde]	20.54±1.43[abc]	21.34±1.19[ab]	14.81±0.95[efgh]	16.49±0.42[efgh]	17.89±0.30[de]	20.26±0.81[bcd]	23.12±2.16[a]	22.96±0.94[ab]	12.92±0.51[i]	14.71±0.30[ghi]	14.52±0.91[hi]
1-Butanol	1175	1179	0.46±0.01[f]	0.78±0.08[abc]	0.75±0.06[abc]	0.89±0.11[a]	0.52±0.03[ghi]	0.67±0.07[bcde]	0.66±0.04[bcde]	0.69±0.11[abcd]	0.83±0.13[abc]	0.86±0.10[ab]	0.64±0.05[cdef]	0.74±0.04[abc]	0.80±0.11[abc]
2-Methyl-1-butanol	1217	1229	20.12±3.51[f]	35.98±2.56[ab]	36.82±1.69[a]	35.27±2.54[abc]	19.81±1.11[f]	23.63±1.07[f]	24.68±0.84[ef]	33.17±1.83[abcd]	35.43±1.14[ab]	36.44±1.03[a]	34.08±1.42[abcd]	35.11±2.07[abc]	38.21±1.40[a]
Isoamyl alcohol	1225	1231	62.85±8.56[e]	84.83±6.57[abcd]	88.67±5.34[ab]	91.75±3.95[ab]	75.78±3.73[cd]	75.41±1.46[cd]	73.98±2.43[d]	89.14±2.07[ab]	91.71±1.46[ab]	94.12±2.05[a]	80.24±3.48[bcd]	88.52±2.97[ab]	90.22±2.74[ab]
1-Hexanol	1356	1363	0.06±0.01[g]	0.07±0.01[fg]	0.08±0.01[def]	0.07±0.01[def]	0.08±0.01[def]	0.09±0.01[cde]	0.11±0.01[b]	0.07±0.01[efg]	0.07±0.01[efg]	0.14±0.01[a]	0.08±0.01[def]	0.10±0.01[bc]	0.10±0.01[bc]
2-Ethyl-1-hexanol	1492	1494	0.04±0.01[bc]	0.05±0.01[b]	0.04±0.01[c]	0.04±0.01[bc]	ND	ND	ND	ND	ND	ND	0.07±0.01[a]	ND	ND
2-Methoxy phenol	1860	1865	ND	ND	ND	0.23±0.01[de]	ND	0.23±0.01[de]	0.87±0.02[a]	ND	0.23±0.01[de]	0.24±0.01[d]	ND	0.39±0.02[c]	0.51±0.01[b]
Benzyl alcohol	1885	1883	0.04±0.01[j]	0.08±0.01[defg]	0.07±0.01[fghi]	0.08±0.01[efgh]	0.05±0.01[hij]	0.09±0.01[cdef]	0.10±0.01[cde]	0.05±0.00[hij]	0.11±0.01[c]	0.11±0.01[cd]	0.15±0.01[b]	0.22±0.04[a]	0.24±0.02[a]
Phenylethyl alcohol	1922	1920	7.91±0.16[bcde]	7.56±0.55[bcde]	7.40±0.24[de]	7.35±0.24[e]	7.46±0.80[cde]	8.31±0.91[abcde]	9.39±0.18[a]	8.64±0.36[abcd]	8.54±0.18[bcde]	8.09±0.40[bcde]	8.36±0.67[abcde]	8.18±0.14[abcde]	7.74±0.31[bcde]
Aldehydes															
Acetaldehyde	744	747	0.34±0.02[f]	0.77±0.03[bcde]	0.86±0.02[abc]	0.97±0.01[a]	0.63±0.03[e]	0.64±0.05[e]	0.66±0.01[de]	0.70±0.05[cde]	0.78±0.07[bcde]	0.88±0.02[abc]	0.73±0.09[cde]	0.72±0.02[cde]	0.94±0.19[ab]
Benzaldehyde	1520	1521	0.05±0.01[k]	0.11±0.01[ij]	0.13±0.01[efghi]	0.14±0.01[de]	0.15±0.01[d]	0.17±0.01[c]	0.24±0.01[b]	0.12±0.00[ghij]	0.13±0.01[efgh]	0.18±0.01[c]	0.14±0.01[defg]	0.26±0.01[ab]	0.26±0.01[a]
2,4-Nonadienal	1710	1701	ND	ND	ND	ND	0.08±0.01[c]	0.09±0.01[c]	0.11±0.01[b]	ND	ND	ND	0.09±0.01[c]	0.16±0.01[a]	ND
Esters															
Ethyl formate	825	828	ND	ND	ND	0.50±0.01[e]	0.09±0.01[g]	0.30±0.02[f]	0.88±0.01[a]	0.09±0.01[g]	0.40±0.03[de]	0.45±0.02[d]	0.10±0.01[g]	0.63±0.04[c]	0.72±0.08[b]
Ethyl acetate	885	896	12.79±1.42[h]	33.33±1.54[fg]	30.97±0.62[g]	34.46±0.94[efg]	33.21±3.13[fg]	33.61±1.50[fg]	36.17±0.62[def]	37.98±2.85[cde]	38.00±2.46[cde]	39.73±1.14[bcd]	40.17±0.93[bc]	42.01±2.71[b]	45.87±1.35[a]
Isoamyl acetate	1126	1125	9.57±0.55[a]	4.65±0.4[efg]1	5.14±0.33[def]	5.24±0.17[def]	4.65±0.15[efg]	6.38±0.31[c]	7.29±0.49[b]	4.36±0.35[fg]	5.76±0.18[cd]	6.01±0.20[cd]	3.84±0.09[g]	5.30±0.33[de]	5.28±0.13[de]
Ethyl valerate	1131	1133	0.04±0.01[f]	ND	ND	ND	ND	ND	ND	ND	0.07±0.01[d]	0.06±0.01[e]	0.10±0.00[c]	0.11±0.01[b]	0.11±0.01[b]
Ethyl heptanoate	1332	1329	0.24±0.01[c]	0.37±0.01[b]	0.36±0.01[b]	0.35±0.01[b]	0.36±0.01[b]	0.36±0.01[b]	0.36±0.02[b]	0.35±0.02[b]	0.37±0.01[b]	0.37±0.01[ab]	0.35±0.02[b]	0.40±0.02 [a]	0.35±0.01[b]
Isobutyl caproate	1347	1347	ND	0.07±0.01[cd]	0.08±0.01[bc]	0.09±0.01[b]	0.05±0.01[de]	ND	ND	0.09±0.01[b]	0.09±0.01[b]	0.11±0.01[a]	0.09±0.01[b]	0.07±0.01[cd]	ND
Ethyl lactate	1353	1353	0.20±0.02[f]	0.29±0.0[cde]1	0.30±0.03[cde]	0.32±0.01[cde]	0.26±0.02[e]	0.28±0.01[cde]	0.28±0.04[cde]	0.30±0.02[cde]	0.34±0.02[c]	0.33±0.04[cd]	0.43±0.01[b]	0.57±0.03[a]	0.49±0.04[b]
Ethyl caprylate	1435	1432	21.6±0.95[f]	28.64±0.50[bcd]	27.73±0.09[bcd]	27.11±0.30[cd]	27.86±0.63[bcd]	29.05±1.41[bcd]	28.20±0.31[bcd]	27.7±1.91[bcd]	31.64±1.04[a]	27.57±0.14[bcd]	27.75±1.60[bcd]	24.31±0.29[e]	26.99±0.97[d]
Isoamyl caproate	1453	1456	0.14±0.01[f]	0.18±0.01[de]	0.19±0.01[cde]	0.16±0.03[ef]	0.22±0.01[bc]	0.22±0.01[b]	0.30±0.01[a]	0.22±0.01[b]	0.22±0.01[b]	0.20±0.01[bcd]	0.23±0.02[b]	ND	ND
Ethyl nonanoate	1526	1534	0.52±0.02[h]	1.09±0.07[abcd]	1.17±0.04[ab]	1.14±0.03[abc]	0.97±0.04[def]	1.02±0.02[bcd]	1.16±0.15[ab]	0.99±0.07[cde]	1.21±0.08[a]	1.06±0.03[abcd]	1.01±0.09[bcde]	0.84±0.05[fg]	0.85±0.03[efg]
Ethyl 2-hydroxyisocaproate	1545	1548	0.06±0.01[d]	0.11±0.01[a]	0.11±0.01[a]	0.11±0.01[ab]	0.10±0.01[abc]	0.09±0.01[abc]	0.09±0.01[c]	0.09±0.01[abc]	0.10±0.01[a]	0.09±0.01	0.08±0.01[cd]	0.09±0.01[bc]	0.10±0.01[abc]
Ethyl caprate	1643	1638	11.47±0.17[g]	26.87±0.64[a]	25.27±0.30[ab]	23.88±0.48[bcde]	24.42±0.83[bcd]	25.28±1.38[ab]	25.11±0.14[ab]	24.70±0.58[abc]	24.14±1.17[bcde]	22.59±0.92[cdef]	23.81±1.27[bcde]	22.65±1.17[cdef]	22.19±1.46[def]
Isoamyl caprylate	1664	1659	ND	ND	ND	ND	ND	ND	ND	ND	ND	ND	ND	ND	ND
Diethyl succinate	1681	1679	0.15±0.01[h]	0.23±0.02[g]	0.33±0.02[ef]	0.41±0.01[cd]	0.21±0.01[g]	0.40±0.01[cd]	0.47±0.04[b]	0.30±0.02[f]	0.35±0.03[ef]	0.36±0.01[de]	0.22±0.02[g]	0.50±0.02[ab]	0.52±0.02[a]
Ethyl undecanoate	1737	1741	ND	0.03±0.00[a]	0.02±0.00[c]	ND	0.03±0.00[b]	ND	ND	ND	ND	ND	ND	ND	ND
Isobutyl decanoate	1751	1755	ND	0.05±0.01[bc]	0.05±0.01[b]	0.04±0.01[cd]	0.05±0.01[bc]	0.05±0.01[bc]	0.06±0.01[b]	0.04±0.01[cd]	0.05±0.01[cd]	0.07±0.01[a]	0.04±0.00[cd]	0.04±0.01[cd]	0.05±0.00[bc]
Methyl salicylate	1755	1778	0.16±0.01[b]	0.07±0.01[ij]	0.06±0.01[jk]	0.06±0.01[jk]	0.08±0.01[hij]	0.14±0.01[c]	0.22±0.01[a]	ND	ND	0.08±0.01[hi]	0.05±0.01[k]	0.09±0.01[gh]	0.14±0.01[cd]
Phenylethyl acetate	1825	1820	4.30±0.16[h]	6.05±0.16[abc]	6.03±0.23[abc]	5.86±0.18[abc]	4.90±0.31[fg]	6.11±0.28[ab]	6.19±0.12[a]	5.22±0.32[defg]	5.74±0.50[abcd]	6.00±0.18[abc]	4.77±0.25[gh]	6.08±0.06[abc]	6.07±0.16[abc]
Ethyl laurate	1847	1845	0.32±0.01[k]	2.33±0.08[a]	2.11±0.06[b]	2.04±0.02[bc]	1.94±0.11[bcd]	1.65±0.09[fgh]	1.78±0.01[defg]	1.87±0.07[cde]	1.82±0.12[def]	1.38±0.13[ij]	1.71±0.16[efg]	1.71±0.09[efg]	1.44±0.09[ij]
Ethyl myristate	2043	2052	0.07±0.01[k]	0.61±0.01[a]	0.57±0.05[ab]	0.57±0.05[ab]	0.51±0.03[bc]	0.48±0.05[cd]	0.40±0.01[ef]	0.43±0.05[de]	0.41±0.03[ef]	0.40±0.02[ef]	0.29±0.03[g]	0.32±0.03[g]	0.32±0.04[g]
Ethyl palmitate	2243	2259	0.12±0.01[f]	0.87±0.05[a]	0.80±0.04[ab]	0.79±0.02[ab]	0.71±0.06[bc]	0.68±0.05[c]	0.72±0.03[bc]	0.79±0.07[ab]	0.78±0.02[ab]	0.72±0.04[bc]	0.70±0.07[bc]	0.48±0.02[fg]	0.48±0.02[fg]

Furans														
2-Amylfuran	1230	1220	ND	ND	ND	ND	ND	ND	ND	ND	ND	ND	0.40±0.02^{c}	0.43±0.01^{b}	0.48±0.01^{a}
Furfural	1466	1466	ND	0.95±0.03^{g}	7.23±0.36^{d}	12.39±0.13^{b}	ND	4.17±0.22^{f}	5.79±0.08^{e}	0.88±0.08^{g}	8.71±0.45^{c}	9.09±0.81^{c}	ND	3.35±0.23^{f}	5.84±0.31^{e}
Benzofuran	1489	1500	ND	ND	ND	0.11±0.01^{bcd}	ND	0.10±0.01^{de}	0.19±0.01^{a}	ND	0.11±0.01^{cd}	0.09±0.01^{ef}	ND	0.12±0.01^{b}	0.18±0.01^{a}
2-Acetylfuran	1510	1504	ND	ND	ND	0.08±0.01^{a}	ND	ND	ND	ND	ND	0.07±0.01^{b}	ND	ND	ND
5-Methyl furfural	1578	1574	ND	0.02±0.00^{i}	2.23±0.05^{f}	4.31±0.03^{b}	ND	1.75±0.03^{g}	2.87±0.02^{e}	0.02±0.00^{i}	3.58±0.18^{d}	3.82±0.13^{c}	ND	1.42±0.07^{h}	1.77±0.11^{g}
2-Methylbenzofuran	1589	1591	ND	ND	0.21±0.02^{ef}	0.23±0.01^{e}	ND	0.20±0.01^{ef}	0.62±0.05^{a}	ND	0.35±0.01^{c}	0.33±0.03^{c}	ND	0.23±0.01^{e}	0.42±0.02^{b}
Ethyl 2-furoate	1621	1623	ND	ND	ND	ND	0.03±0.00^{d}	ND	ND	ND	ND	ND	0.03±0.00^{e}	0.12±0.01^{a}	ND
Hydrocarbons															
2,4-Dimethylheptane	797	813	0.37±0.03^{bcd}	0.25±0.01^{f}	0.43±0.03^{ab}	0.37±0.01^{bcd}	0.42±0.03^{ab}	0.33±0.03^{de}	0.25±0.01^{f}	0.35±0.03^{cde}	0.35±0.02^{cde}	0.30±0.01^{ef}	0.39±0.04^{abcd}	0.38±0.03^{abcd}	0.38±0.04^{bcd}
4-Methyloctane	823	859	1.79±0.26^{a}	0.70±0.04^{fg}	0.97±0.06^{de}	0.50±0.01^{g}	0.60±0.02^{fg}	0.83±0.08^{ef}	1.18±0.03^{cd}	1.22±0.09^{c}	1.21±0.13^{cd}	1.33±0.11^{bc}	1.20±0.05^{cd}	1.19±0.06^{cd}	1.26±0.02^{bc}
1,2,4,5-Tetramethylbenzene	1417	1413	0.12±0.01^{b}	0.05±0.01^{f}	0.06±0.01^{ef}	0.06±0.01^{ef}	0.06±0.01^{de}	0.07±0.01^{de}	0.14±0.01^{a}	0.07±0.00^{d}	0.07±0.00^{de}	0.07±0.01^{cd}	0.05±0.01^{f}	ND	ND
Lactones															
Butyrolactone	1635	1631	ND	ND	0.02±0.00^{h}	0.02±0.00^{h}	0.05±0.01^{def}	0.05±0.01^{de}	0.05±0.01^{de}	ND	0.04±0.01^{fg}	0.04±0.01^{ef}	0.03±0.00^{g}	0.08±0.00^{a}	0.07±0.00^{a}
trans-Whiskey lactone	1973	1892	ND	0.29±0.01^{d}	0.32±0.00^{d}	0.29±0.02^{d}	ND	ND	ND	0.15±0.03^{g}	0.19±0.02^{f}	0.22±0.01^{e}	ND	ND	ND
Oaklactone	1971	1966	ND	0.24±0.02^{g}	0.30±0.01^{f}	0.32±0.01^{ef}	ND	ND	ND	0.39±0.01^{cd}	0.50±0.03^{b}	0.55±0.02^{a}	ND	ND	ND
Phenols															
Phenol	2004	2010	ND	ND	ND	ND	ND	ND	0.08±0.00^{a}	ND	ND	ND	ND	0.07±0.01^{a}	ND
Eugenol	2172	2175	ND	ND	ND	0.07±0.01^{de}	0.08±0.01^{cd}	0.12±0.01^{b}	0.27±0.03^{a}	0.04±0.01^{f}	0.10±0.01^{bc}	0.10±0.01^{bc}	ND	0.10±0.00^{bc}	0.10±0.01^{bc}

Supplementary Table 1. (continued)

Compounds	KI^{L}	KI^{M}	Relative Concentration (μg/mL) ^{N}						Aroma character
			SIN-1	SIN -3	SIN -5	JOL-1	JOL-3	JOL-5	
Acetals									
1-Ethoxy-1-pentyloxyethane	1104	1111	0.06±0.00^{bcd}	0.07±0.01^{bcd}	0.07±0.00^{bcd}	0.06±0.01^{cd}	0.06±0.00^{bcd}	0.07±0.01^{abcd}	
Acids									
Acetic acid	1461	1461	0.90±0.11^{ij}	4.15±0.79^{de}	4.81±0.22^{bcd}	1.03±0.13^{hij}	2.71±0.42^{fg}	3.81±0.42^{def}	acid, fruit, pungent, sour, vinegar
Propanoic acid	1556	1559	ND	ND	ND	ND	ND	ND	fat, pungent, rancid, silage, soy
Butanoic acid	1663	1658	ND	ND	ND	ND	ND	ND	butter, cheese, rancid, sour, sweat
Alcohols									
Ethanol	937	941	238.92±2.76^{bcde}	257.14±14.52^{bcd}	257.79±2.23^{bcd}	245.09±5.26^{bcde}	242.09±17.59^{bcde}	245.36±6.77^{bcde}	alcohol, floral, ripe apple, sweet
1-Propanol	1061	1069	1.83±0.12^{bcd}	1.94±0.09^{bc}	1.95±0.15^{b}	1.28±0.05^{fg}	1.44±0.20^{defg}	1.37±0.2^{efg}	candy, must, pungent, ripe fruit
Isobutyl alcohol	1108	1119	15.94±0.64^{efgh}	17.08±1.19^{efgh}	17.51±0.65^{efg}	15.03±1.25^{fghi}	16.97±2.16^{efgh}	17.69±0.68^{def}	apple, cocoa, malt
1-Butanol	1175	1179	0.82±0.07^{abc}	0.80±0.13^{abc}	0.73±0.11^{abc}	0.48±0.02^{ef}	0.81±0.09^{abc}	0.75±0.06^{abc}	alcohol, fruit, medicine, phenol
2-Methyl-1-butanol	1217	1229	34.35±2.47^{abcd}	36.02±1.02^{ab}	37.13±0.93^{a}	29.74±4.75^{cde}	30.43±4.07^{bcd}	28.90±1.67^{de}	banana, green, malt, medicine
Isoamyl alcohol	1225	1231	88.48±4.43^{ab}	89.82±5.19^{ab}	89.64±3.24^{ab}	81.82±5.84^{bcd}	85.52±7.48^{abc}	88.32±3.70^{ab}	banana, cocoa, floral, fusel, nail polish
1-Hexanol	1356	1363	0.09±0.01^{cde}	0.08±0.01^{cde}	0.09±0.01^{cde}	0.08±0.01^{def}	0.09±0.01^{cd}	0.07±0.01^{defg}	flower, fruit, green, herb, wood
2-Ethyl-1-hexanol	1492	1494	ND	ND	0.04±0.01^{bc}	ND	ND	ND	citrus, green, oil, rose
2-Methoxy phenol	1860	1865	ND	0.13±0.01^{g}	0.20±0.01^{f}	ND	0.13±0.01^{g}	0.22±0.01^{ef}	bacon, medicine, phenol, smoke, wood
Benzyl alcohol	1885	1883	0.04±0.00^{ij}	0.05±0.01^{hij}	0.05±0.00^{hij}	0.05±0.00^{ij}	0.06±0.00^{ghij}	0.05±0.00^{hij}	almond, boiled cherries, floral, moss, roasted bread
Phenylethyl alcohol	1922	1920	8.72±0.37^{abc}	8.44±0.31^{abcde}	8.17±0.33^{abcde}	8.84±0.65^{ab}	8.83±0.93^{ab}	8.18±0.34^{abcde}	fruit, honey, rose, sweet apple, wine
Aldehydes									
Acetaldehyde	744	747	0.75±0.02^{cde}	0.77±0.10^{bcde}	0.85±0.03^{abc}	0.73±0.07^{cde}	0.84±0.08^{abcd}	0.87±0.15^{abc}	floral, fruit, green apple, sweet
Benzaldehyde	1520	1521	0.11±0.01^{hij}	0.15±0.01^{de}	0.14±0.01^{def}	0.11±0.01^{j}	0.12±0.01^{fghij}	0.13±0.01^{efg}	bitter almond, burnt sugar, cherry, malt, roasted pepper

2,4-Nonadienal	17101701	ND	ND	ND	ND	ND	ND	cereal, deep fried, fat, unpleasant, watermelon
Esters								
Ethyl formate	825 828	0.12±0.01^{g}	0.46±0.05^{d}	0.58±0.01^{c}	0.10±0.01^{g}	0.27±0.02^{f}	0.42±0.06^{de}	ethereal, pungent
Ethyl acetate	885 896	31.58±1.06^{g}	31.76±0.8^{g}	33.48±0.73^{fg}	31.81±1.50^{g}	30.48±1.08^{g}	34.24±0.62^{efg}	balsamic, contact glue, grape, pineapple, sweet
Isoamyl acetate	11261125	5.17±0.29^{def}	5.47±0.45^{de}	5.70±0.21^{cd}	4.58±0.23^{efg}	5.11±0.75^{def}	5.28±0.33^{de}	apple, banana, glue, pear, sweet
Ethyl valerate	11311133	0.12±0.01^{a}	0.06±0.01^{de}	0.07±0.01^{de}	ND	ND	ND	apple, dry fish, herb, nut, yeast
Ethyl heptanoate	13321329	0.36±0.02^{b}	0.35±0.02^{b}	0.37±0.01^{ab}	0.38±0.01^{ab}	0.38±0.01^{ab}	0.35±0.01^{b}	brandy, fruit, wine
Isobutyl caproate	13471347	0.07±0.01^{cd}	0.07±0.01^{cd}	0.07±0.01^{cd}	0.05±0.00^{e}	0.06±0.00^{cde}	0.08±0.01^{bc}	green, plastic, spice
Ethyl lactate	13531353	0.29±0.03^{cde}	0.27±0.03^{de}	0.29±0.02^{cde}	0.27±0.01^{de}	0.33±0.04^{cd}	0.27±0.01^{de}	butter, cream, floral, fruit, sweet
Ethyl caprylate	14351432	29.50±0.08^{b}	27.53±1.28^{bcd}	28.47±0.23^{bcd}	29.41±0.64^{bc}	28.24±1.00^{bcd}	27.06±0.85^{cd}	apricot, banana, brandy, pear, pineapple
Isoamyl caproate	14531456	0.23±0.01^{b}	0.20±0.01^{bcd}	0.15±0.03^{f}	0.23±0.01^{b}	0.21±0.01^{bcd}	0.19±0.01^{cde}	anise, caramel, fruit, spice, yeast
Ethyl nonanoate	15261534	1.04±0.05^{bcd}	1.15±0.10^{abc}	1.16±0.03^{ab}	1.02±0.06^{bcd}	1.10±0.08^{abcd}	0.80±0.05^{g}	fruit
Ethyl 2-hydroxyisocaproate	15451548	0.10±0.01^{abc}	0.08±0.01^{cd}	0.10±0.01^{abc}	0.10±0.00^{abc}	0.10±0.0^{abc}	0.09±0.01^{abc}	black currant, fruit
Ethyl caprate	16431638	23.94±0.42^{bcde}	22.11±0.78^{def}	22.15±0.42^{def}	24.11±0.87^{bcde}	21.84±1.91^{f}	21.18±0.19^{ef}	brandy, burnt, grape, nut, pear
Isoamyl caprylate	16641659	ND	ND	0.73±0.05^{a}	ND	ND	ND	baked apple
Diethyl succinate	16811679	0.33±0.02^{ef}	0.40±0.04^{cd}	0.46±0.01^{b}	0.24±0.02^{g}	0.41±0.01^{c}	0.41±0.01^{c}	cotton, cream, floral, fruit, wine
Ethyl undecanoate	17371741	ND	ND	ND	ND	ND	ND	coconut, cognac, fruit
Isobutyl decanoate	17511755	0.04±0.00^{d}	0.04±0.01^{cd}	0.03±0.00^{d}	0.04±0.00^{cd}	0.05±0.00^{bc}	0.04±0.00^{cd}	baked apple
Methyl salicylate	17551778	0.08±0.01^{hi}	0.09±0.01^{gh}	0.12±0.01^{de}	0.11±0.01^{ef}	0.10±0.01^{fg}	0.16±0.01^{b}	almond, caramel, medicine, peppermint, sharp
Phenylethyl acetate	18251820	5.15±0.02^{defg}	5.51±0.34^{cde}	5.67±0.07^{bcde}	5.08±0.08^{efg}	5.59±0.38^{abcde}	5.48±0.12^{cdef}	floral, fruit, honey, rose, tobacco
Ethyl laurate	18471845	1.59±0.01^{ghi}	1.47±0.09^{hij}	1.50±0.04^{hij}	1.57±0.04^{ghi}	1.41±0.13^{ij}	1.36±0.03^{j}	floral, fruit, green apple, leaf, nut
Ethyl myristate	20432052	0.35±0.03^{fg}	0.35±0.02^{efg}	0.36±0.01^{efg}	0.36±0.01^{efg}	0.34±0.02^{fg}	0.29±0.01^{g}	ether, nut, oil, pleasant, soap
Ethyl palmitate	22432259	0.62±0.04^{cde}	0.58±0.02^{de}	0.66±0.01^{cd}	0.56±0.03^{ef}	0.63±0.05^{cde}	0.43±0.01^{e}	wax
Furans								
2-Amylfuran	12301220	ND	ND	ND	ND	ND	ND	butter, floral, fruit, green bean
Furfural	14661466	0.97±0.05^{g}	12.65±1.53^{b}	14.89±0.17^{a}	0.71±0.10^{g}	6.57±0.28^{de}	9.69±1.04^{c}	almond, baked potatoes, bread, candy, floral
Benzofuran	14891500	ND	0.09±0.01^{f}	0.11±0.01^{bc}	ND	0.06±0.00^{g}	0.11±0.01^{cd}	rotten
2-Acetylfuran	15101504	ND	0.09±0.01^{a}	ND	ND	ND	0.06±0.01^{b}	balsamic, cocoa, coffee, smoke, tobacco
5-Methyl furfural	15781574	0.02±0.00^{i}	4.29±0.34^{b}	4.57±0.04^{a}	ND	1.99±0.04^{g}	3.51±0.16^{d}	almond, caramel, cooked, roasted garlic, spice
2-Methylbenzofuran	15891591	ND	0.17±0.03^{fg}	0.28±0.01^{d}	ND	0.09±0.01^{h}	0.15±0.01^{g}	
Ethyl 2-furoate	16211623	0.03±0.00^{de}	0.03±0.00^{de}	0.07±0.00^{b}	ND	ND	0.06±0.00^{c}	caramel
Hydrocarbons								
2,4-Dimethylheptane	797 813	0.44±0.01^{a}	0.40±0.03^{abc}	0.37±0.01^{bcd}	0.38±0.01^{abcd}	0.35±0.01^{cde}	0.39±0.04^{abc}	
4-Methyloctane	823 859	1.90±0.11^{a}	1.50±0.07^{b}	1.48±0.11^{b}	1.85±0.10^{a}	1.76±0.13^{a}	1.50±0.09^{b}	
1,2,4,5-Tetramethylbenzene	14171413	0.07±0.00^{cd}	0.08±0.01^{c}	0.06±0.00^{de}	0.07±0.00^{cd}	0.06±0.01^{de}	0.07±0.00^{cd}	rancid, sweet
Lactones								
Butyrolactone	16351631	0.05±0.00^{bcd}	0.06±0.00^{b}	0.06±0.00^{b}	0.05±0.00^{cd}	0.06±0.00^{bc}	0.06±0.01^{bcd}	caramel, cheese, fruit, roasted nut, sweat
trans-Whiskey lactone	19731892	0.10±0.01^{h}	0.44±0.02^{b}	0.47±0.03^{a}	0.22±0.01^{e}	0.30±0.01^{d}	0.35±0.01^{c}	coconut, flower, sick, sweet, wood
Oaklactone	19711966	0.40±0.02^{c}	0.41±0.03^{c}	0.49±0.02^{b}	0.32±0.02^{ef}	0.35±0.03^{de}	0.40±0.02^{c}	butter, cocoa, coconut, sweet
Phenols								
Phenol	20042010	ND	ND	0.06±0.00^{b}	ND	ND	ND	medicine, sharp, smoke, spice
Eugenol	21722175	ND	0.07±0.00^{de}	0.06±0.00^{e}	ND	0.08±0.01^{cd}	0.10±0.01^{bc}	burnt, clove, smoke, spice

Odor character from https://www.vcf-online.nl/VcfHome.cfm. All values are represented by mean ± standard deviation (*n* = 3). [L] Kovats index calculated using *n*-alkanes for the DB-WAX column. [M] Kovats index reported from NIST available in http://webbook.nist.gov/chemistry/cas-ser.html for DB-WAX columns or equivalents. [N] Relative concentration; the concentrations calculated as equivalents relative to the internal standard (4.878 μg/mL of linalool), ND; not detected
